# Defects on the Surface of Ti-Doped MgAl_2_O_4_ Nanophosphor

**DOI:** 10.1186/s11671-017-2288-1

**Published:** 2017-09-19

**Authors:** Jaehyuk Lim, Yongseon Kim, Sungdae Kim, Youngwoon Kim, Shinhoo Kang

**Affiliations:** 1 Samsung Electronics, 95 Samsung2-ro, Giheung-gu, Yongin, Gyeonggi-do 446-811 South Korea; 2 Department of Materials Science and Engineering, Inha University, Incheon, 402-751 South Korea; 3 Korea Institute of Materials Science(KIMS), 797 Changwondaero, Seongsan-gu, Changwon, Gyeongsangnam-do 642-831 South Korea; 4 Department of Materials Science and Engineering, Seoul National University, 1 Gwanak-ro, Gwanak-ku, Seoul, 151-742 South Korea

## Abstract

Ti-doped nano MgAl_2_O_4_ for white emission was synthesized by combustion method. Extrinsic Schottky defects, Al vacancies and Ti^4+^ dopant in Al sites, which are considered to be responsible for bluish-white emission, were observed by STEM on the surface of Ti-doped nano MgAl_2_O_4_ powder. The stabilities of the Schottky defect associates, (Ti_Al_^·^–V_Al_′′′)′′, were demonstrated by DFT calculation. The emission behavior was interpreted with these results.

## Background

The transition from bulk or micron to nanosize domain greatly affects a material, altering, for example, its mechanical, optical, and electrical properties [1–6]. These changes are mostly attributed to the size and associating non-equilibrium structure. An example is the unique phosphorescence and emission properties achievable by nanoparticles [2, 7]. The emission properties of nanophosphors can be modulated by doping, in addition to the quantum confinement effects described [8, 9]. The charge valence of a dopant and the site in the structure that it occupies generally affect the emission properties of a phosphor. Dopants can often be located in a nanophosphor at sites (e.g., on a particle’s surface) other than the usual sites in a micron-sized phosphor. Thus, the surfaces of nanoparticles become important sites for dopants that do not normally occupy such sites in bulk or micron systems. The changes in the emission behavior have been reported due to the occupation site, which is associated with other defects [8, 9].

Pure MgAl_2_O_4_ has an intrinsic defect of an Mg^2+^ vacancy, V_Mg_′′, which is the center of a red emission at 720 nm. Strong blue emission is observed from single crystals of Ti-doped MgAl_2_O_4_; the disappearance of the red emission is attributed to charge compensation through the addition of Ti^4+^ [10, 11]. However, in our previous work, we found that Ti-doped micron-sized MgAl_2_O_4_ powder heat-treated in air produced a white emission [12]. The difference was explained via the occurrence of red and green emissions in addition to the blue observed from Ti-doped MgAl_2_O_4_ single crystals. Our previous work [12] also simulated the mechanism for the red emission via intrinsic Schottky defect associate, (V_O_^··^–V_Mg_′′)^x^. The present work reports the visual observation of extrinsic Schottky defects on the surface of Ti-doped MgAl_2_O_4_ nanopowder and relates it to the difference in the emission spectra between micron and nano systems.

## Methods

Mg(NO_3_)_2_·6H_2_O (Mg nitrate; 2.46 g, Aldrich), Al(NO_3_)_3_·9H_2_O (Al nitrate; 7.246 g, Aldrich), CO(NH_2_)_2_ (urea; 5.231 g, Aldrich), and C_10_H_14_O_5_Ti (Ti oxy-acetyl-acetonate; 0.1 g, Aldrich,) were used as starting materials for the synthesis of Ti-doped nano MgAl_2_O_4_. Mg nitrate and Al nitrate were used in a 1:2 molar ratio in the synthesis, and 2 mol% Ti doping was provided by Ti oxy-acetyl-acetonate. The starting materials were dissolved in deionized water, and the mixture was homogenized by stirring, before the water was evaporated on a hot plate. The remaining mixture was placed in an alumina crucible and fired at 500 for 1 h in air.

The phases of the synthesized nanopowder were analyzed by X-ray diffractometry (XRD; Rigaku), and photoluminescence properties were measured by fluorescence spectrophotometry (PSI, PL Darsa pro-5000 system) using monochromated 260 and 360 nm light from a Xe lamp. Powder morphology and size were observed by high-resolution transmission electron microscopy (TEM; JEOL, JEM-2100F). Images of the Ti dopant and the Al vacancies were also obtained by high-resolution scanning TEM (HR-STEM; JEOL, JEM-2100F).

First principles density functional theory (DFT) calculations were performed based on the generalized gradient approximation of Perdew–Burke–Ernzerhof and projector-augmented plane wave pseudopotentials implemented in the Vienna ab initio simulation package (VASP) [13–15] with an energy cutoff of 500 eV and a self-consistency field convergence of 10^−5^ eV. The stabilities of various defect associates were examined by calculation to investigate their dependence upon the positions of the dopants and vacancies and the relative distances between them.

The surface energy of the (100) surface plane in the MgAl_2_O_4_ crystal was calculated; its variation with the Ti site was also examined. The unit cell—whose crystal structure was previously optimized allowing full relaxation of the lattice parameter, crystal shape, and atomic positions—was expanded to a 4 × 1 supercell. {100} surfaces were created by inserting a vacuum slab inside the supercell. Insertion position of the vacuum slab which is of the size of 2 × 1 supercell was varied to examine change of the surface formation energy with the distance between the surface and the Ti dopant. Surface termination with 50% of Mg layer was mainly considered because this was found to be the most stable (1 0 0) surface of MgAl_2_O_4_.

## Results and Discussion

**Fig. 1:**
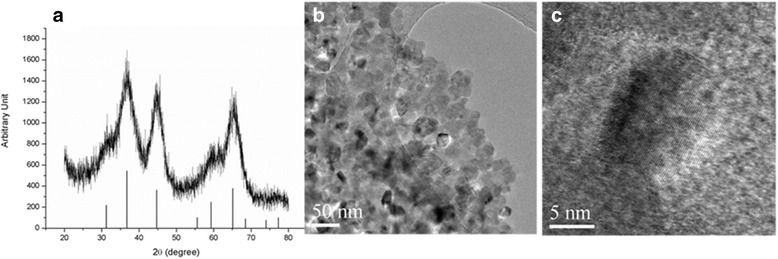
**a** XRD pattern for Ti-doped nano MgAl_2_O_4_ powder synthesized at 500 °C 1 h with reference peaks of MgAl_2_O_4_ from JCPDS and **b**, **c** TEM images of the powder

Figure 1 shows the XRD pattern for Ti-doped MgAl_2_O_4_ nano phosphors with TEM graphs. Figure 1a clearly confirms that MgAl_2_O_4_ was synthesized at 500 °C by the combustion method, given its similarity to the JCPDS XRD pattern for pure MgAl_2_O_4_. The broad peaks indicate the presence of nanocrystallites and are related to the particles of < 20 nm shown in Fig. 1b. In contrast, the Ti-doped micron-sized MgAl_2_O_4_ of our previous work [12] shows high crystallinity, attributed to the high-temperature treatment of the MgAl_2_O_4_ powder (1300 °C for 2 h).

**Fig. 2:**
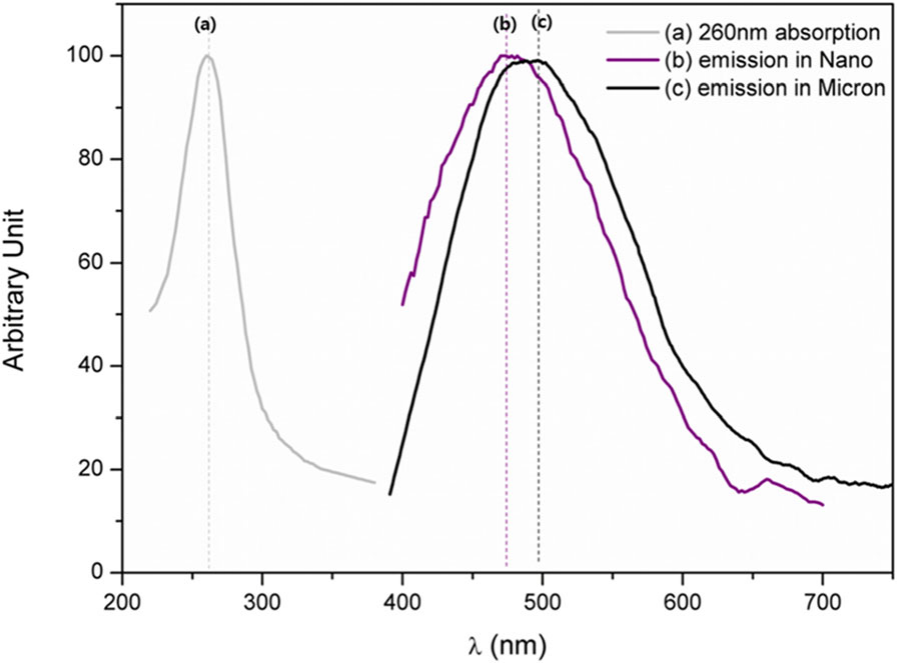
Photoluminescence of Ti-doped MgAl_2_O_4_: **a** 260 nm excitation, **b** nano, and **c** micron [12] powders synthesized at 500 °C, 1 h, and 1300 °C, 2 h, respectively

The photoluminescence emission spectra of Ti-doped MgAl_2_O_4_ show white emission at 260 nm excitation (Fig. 2a) for nano- and micron-sized samples synthesized at 500 °C for 1 h and 1300 °C for 2 h in Fig. 2b, c, respectively. However, the two emission bands result in slightly different colors: that of the nanopowder synthesized at 500 °C is blue-shifted relative to that of the micron-scale powder prepared at 1300 °C. The blue emission of Ti-doped MgAl_2_O_4_ single crystals is attributed to Ti^4+^ in Al (octahedral) sites, which was the only form of Ti ions in the single crystals [10, 11]. However, Ti-doped MgAl_2_O_4_ micron-sized powder was shown to have both Ti^3+^ and Ti^4+^ equally occupying both Al (octahedral) and Mg (tetrahedral) sites [12].

**Fig. 3:**
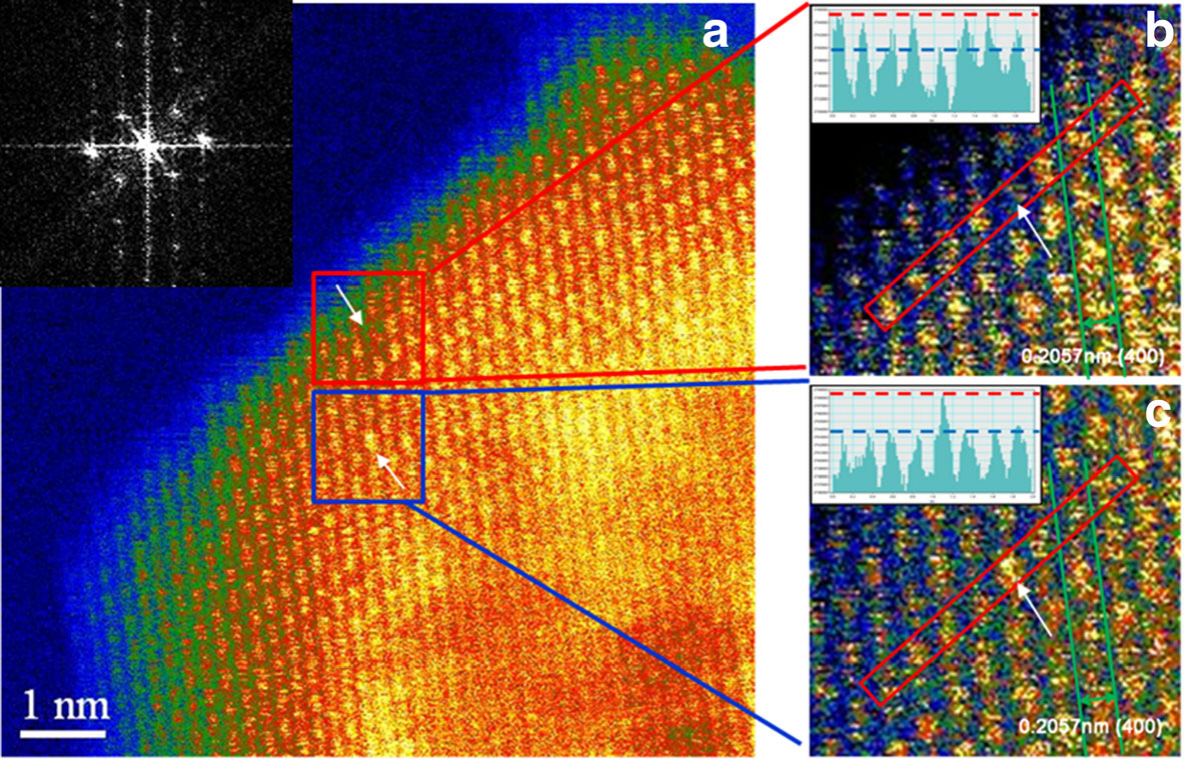
HR-STEM images of Ti-doped nano MgAl_2_O_4_; **a**. The image shows Al^3+^ vacancy and Ti^4+^ dopant in the vicinity with Fourier transformation of the image. **b** The magnified image in red box of (**a**) and Al^3+^vacancy is revealed from STEM image with a corresponding contrast intensity, inset of (**b**). **c** The contrast intensity, inset of (**c**), confirms that Ti^4+^ dopant occupies an Al site. The arrows indicate the locations of Al vacancy and Ti in Al site, respectively

Figure 3a shows an HR-STEM image taken near the surface of Ti-doped nano MgAl_2_O_4_. The magnified image in Fig. 3b shows the distance between the arrays to be 0.2057 nm, which matches well with the (400) planar distance of MgAl_2_O_4_ (0.202 nm). It shows that the atomic arrangement left a relatively dark vacancy among the spots (see arrows in Fig. 3a, b). The minor brightness at the vacancy might have originated from atoms in the lower layers. The defect point is also identified in the contrast intensity plot in the inset, which shows the contrast peaks for the atoms inside the red box of Fig. 3b. The vacancy is clearly shown by the low contrast-intensity of the fifth site from left. To identify the vacancy site, we performed the Fourier transformation of the image in Fig. 3a and found that the beam axis is close to [001] (inset, Fig. 3a). It is noted from the [001] projected view of a MgAl_2_O_4_ crystal that Mg atoms are located independently in the (004) plane, whereas Al and O atoms appear overlapped together in the same plane. Thus, if the fluctuation shown in the contrast intensity is only due to the constituent atoms on the plane, it is more probable for the vacancy to originate from a vacant Al site rather than a vacant Mg site.

In Fig. 3c, the lattice point indicated by an arrow in the red box is much brighter than the others. Considering that Mg and Al atoms cannot be distinguished by z-contrast due to their similar atomic numbers and that it is difficult to detect oxygen atoms owing to its low atomic number, this brighter point is concluded to be due to the Ti dopant. The corresponding contrast intensity plot (inset, Fig. 3c) emphasizes the brighter spot, indicating the presence of an element of higher atomic number, definitely Ti in this system. Ti in an Al site causes displacement error, because its charge valence and ionic radius are different from those of Al^3+^. The brighter atom in the figure appears larger than the others, in agreement with the larger effective ionic radii of Ti^3+^ (0.081 nm) and Ti^4+^ (0.0745 nm) in comparison to that of Al^3+^ (0.0675 nm) [16]. The effective ionic radius of Mg^2+^ is reported as 0.086 nm, which is larger than those of the Ti ions. Thus, we concluded that the defects shown in Fig. 3 (i.e., Fig. 3b, c) are V_Al_′′′ and Ti_Al_^·^, respectively, expecting that Ti^4+^ ions of a smaller size (0.0745 nm) have more chance to take vacant Al sites than Ti^3+^ (0.081 nm).

**Fig. 4:**
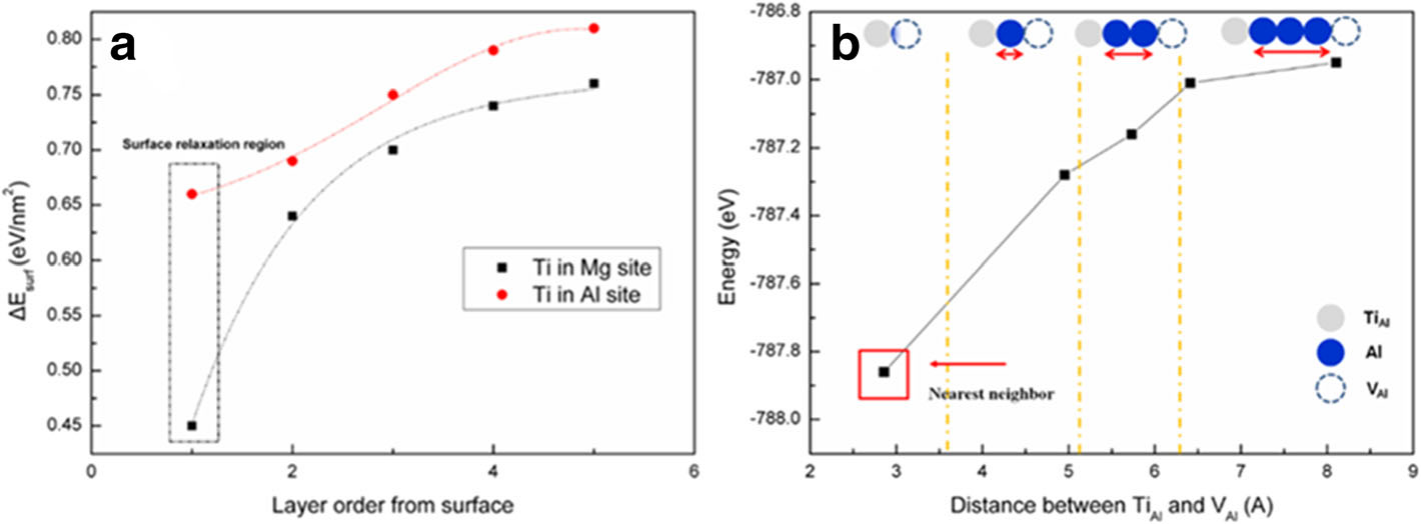
**a** Stabilities of Ti in Mg or Al vacancy site on the surface of MgAl_2_O_4_: the red circle shows surface energy of Ti in Al site and the black dot is that of Ti in Mg site and **b** the binding energy of defect associates, Ti_Al_^·^–V_Al_′′′ as a function of distance

Figure 4a shows the change in the surface energy of a Ti-doped MgAl_2_O_4_ perfect crystal calculated with respect to the position of the dopant. The surface energy, which may be a major factor influencing the formation energy of a nanosystem, decreases as Ti approaches the surface, indicating that the crystal is more stable when Ti is closer to the surface. The result indicates a common trend for Ti at an Al site and Ti at an Mg site; however, the dopant is more stable at an Mg site than at an Al site. This is attributed to the larger effective ionic radius of Mg^2+^(0.086 nm) than either Ti^4+^(0.0745 nm) or Al^3+^(0.0675 nm) [16].Thus, the trend is more probable when Ti-doped MgAl_2_O_4_ has a high crystallinity. However, it might not be always true for a nanosystem of a low crystallinity, at least near the surface region.

DFT calculations were also run to investigate the positioning of the Ti dopant and the Al vacancy. The calculated energy of a Ti_1_Mg_15_Al_31_O_64_ crystal, spinel containing a Ti dopant (Ti_Al_^·^) and an Al vacancy (V_Al_′′′), increases as the dopant and vacancy are moved apart as shown in Fig. 4b. Therefore, greater stability is achieved when the two defects are close to each other and form defect associates such as (Ti_Al_^·^–V_Al_′′′)′′ that are responsible for the blue emission. This result is attributed to the structural stability and the Coulombic force between the two point defects. However, a compromise emerges between these factors and the configurational entropy to stabilize the system at elevated temperature, which results in the two defects being spaced 2–3 atoms apart, as shown in Fig. 3a.

In general, the formation energy of an Al or Mg vacancy is much lower (~4.5 eV) than that of an oxygen interstitial (~ 7.0 eV) in MgAl_2_O_4_ [17, 18]. Also, the formation energy of intrinsic Schottky defects for MgAl_2_O_4_ (4.15 eV/defect) is much lower than those for individual oxides, MgO (7.7 eV) and α-Al_2_O_3_ (4.2–5.1 eV). According to Coulomb estimates, the defect association energies of extrinsic Schottky pairs are smaller than those of intrinsic Schottky pairs in various ionic systems [19]. When Ti-doped MgAl_2_O_4_ is synthesized chemically by combustion method via nucleation and precipitation process, as for the nano system of this study, instead of by solid-state diffusion, the formation of defects and defect associates, including O^2−^ vacancies which are commonly observed in oxide ceramics, would be significantly facilitated on the particle surfaces. Overall results indicate that the defect associates, i.e., (Ti_Al_^·^–V_Al_′′′)′′, prevail on the surface of Ti-doped MgAl_2_O_4_ nanopowders, causing the blue shift in the white emission of nano-powders compared to that of micron powders.

## Conclusions

The substitution of Ti in the Al sites of MgAl_2_O_4_ was observed by HR-STEM. An Al vacancy and Ti dopant were detected near the surface of Ti-doped nano MgAl_2_O_4_. These observations demonstrate the presence of Ti^4+^ in Al sites. The blue shift relative to the spectrum of the micron-scale system is attributed to the presence of more Ti^4+^ ions in Al sites at the surface. It would be energetically more favorable for Ti^4+^ ions to take Mg sites in the spinel structure. However, Ti^4+^ ions tend to take Al sites in the Ti-doped nano MgAl_2_O_4_. This difference in the luminescence of the nanosystem arose from its low crystallinity that is resulted from the low processing temperature.

## Abbreviations

Al nitrate: Al(NO_3_)_3_·9H_2_O
DFT: Density functional theory
HR-STEM: High-resolution scanning TEM
Mg nitrate: Mg(NO_3_)_2_·6H_2_O
PL: Photoluminescence
TEM: Transmission electron microscopy
Ti oxy-acetyl-acetonate: C_10_H_14_O_5_Ti
Urea: CO(NH_2_)_2_
VASP: Vienna ab initio simulation package
XRD: X-ray diffractometry
